# Recent trends on the stent research for blood arteries by bibliometric analysis

**DOI:** 10.1186/2055-7124-18-17

**Published:** 2014-11-06

**Authors:** Sejung Ahn, Jung-Suk Sung, Brad Choi, Hackjoo Kim, Yong Kiel Sung

**Affiliations:** Technology Information Analysis Center, Korea Institute of Science and Technology Information, 66 Hoegi-ro, Seoul, Dongdaemun-gu, 130-741 Korea; Department of Life Science, College of Biosystems, Dongguk University, Seoul, 100-715 Korea; Noanix Corporation, 711 Sejung TechnoValley, 134 Gongdanro, Hoengdeok-gu, Chungju-si, Chungbuk, 361-290 Korea; ReSEAT, Korea Institute of Science and Technology Information, 66 Hoegi-ro, Seoul, Dongdaemun-gu, 130-741 Korea

**Keywords:** Stent, Blood artery, Drug-eluting stent, Blood coagulation, Biomaterials

## Abstract

The research trends on stent for blood arteries are reviewed by bibliometric analysis using 7,790 journal articles published from 1986 to 2013 of the Web of Science database. The bibliometric indicators are applied to analyze the journal article data, which are simple number of publications for selecting key players, citation indicators for measuring qualitative research performance, collaboration indicators for figuring out the degree of international collaboration and keyword mapping for identifying the research trends. The studies of stent for blood arteries are investigated on the basis of the analysis by countries, institutions and topic changing. The leading countries and institutions published many high-quality journal articles with strong international collaboration. In this report, the current status and future of research trends are clearly revealed from the periodic topic changing analysis. The keywords such as ‘drug eluting stent’, ‘stent coated with new polymers’ and ‘drug delivery systems’ have come into the recent stent-related research, which means lots of efforts are under way to overcome the present limitations of the research.

## Introduction

A stent is a small mesh tube to be used to treat weak and narrow arteries. Normal arteries are blood vessels that are able to carry blood away from heart to other parts of human body. However, problems occur sometimes in the arteries of old or sick people. In the case of good chance, a stent is placed in an artery as part of a procedure called percutaneous coronary intervention (PCI). PCI restores blood flow through narrow or blocked arteries of the body. A replaced stent helps support the inner wall of the artery within a certain period of time for few months or years after putting on PCI.

Some stents are coated with polymeric drugs that are slowly and continuously released into the artery. The stents are called drug-eluting stents (DES). Introducing DES, several problems have been reduced dramatically in coronary artery stenting by limiting long-term efficacy [1]. The medicine helps prevent the artery from coming blocked again. Recently, researches have indicated that the reduction in restenosis might have been obtained at the expense of a higher incidence of stent thrombosis, particularly late stent thrombosis [2].

In the present paper, recent progress has been reviewed on the stent coating for heart blood arteries of human body. The research trends of the stent coating for heart blood arteries are reviewed using 7,790 journal articles published from 1986 to 2013 in various aspects.

## Review

The journal articles for analysis were collected using search query made of keywords using Web of Science (WoS) database provided by Thomson Scientific (Philadelphia, PA, USA). The used search query was shown in Table 1. The dataset contains Science Citation Index (SCI) and SCI-Expanded (SCIE) with a document type limited to the ‘article’ reflecting the accurate R&D trends and the search period was set to 1986 ~ 2013. The basic analysis was carried out using the COMPAS (COMPetitive Analysis System) developed by KISTI (Korea Institute of Science and Technology Information) and VantagePoint® provided by Search Technology, Inc. The VOSviewer of CWTS and Netminer 4.0 provided by Cyram are used for the network analysis.

**Table 1:** **Search query for the analysis of research trends on stent for blood arteries**

Search query	Limitation
TS = (stent* and ((bio* near/3 stent*) or “biodegradable polymer*” or “biocompatible polymer*” or “biocompatible material*” or “biomaterial*” or anti-coagulant* or “biomedical coating” or “biomedical implant*” or “drug-eluting” or “drug elut*” or “drug delivery coating system”))	PY = 1986 ~ 2013 document type = ‘article’

**Figure 1:**
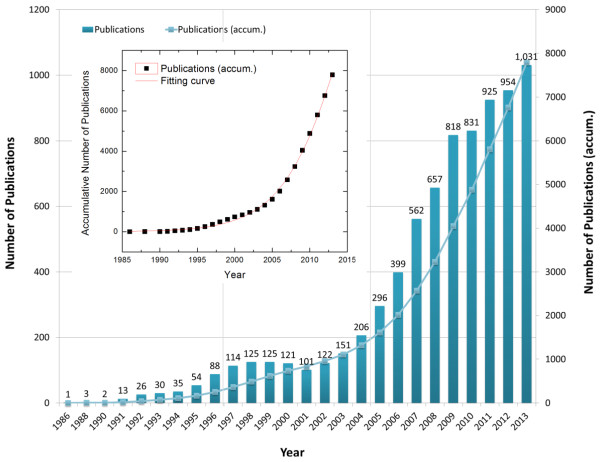
**The number of publications on stent by year; The fitting curves of accumulative number of publications (inset).**

Figure 1 shows the number of publications by the year of 1986 ~ 2013. The annual number of journal articles since 1986 increases steadily in spite of a little fluctuation between 2000 and 2002. However, the accumulative number of publications gradually increases. We can estimate the technology growth level by curve fitting to a suitable model. In this case, the accumulative data are able to be explained by a logistic model. In a logistic model, the general expression for the technology growth level can be written as [3, 4].

**Figure 2:**
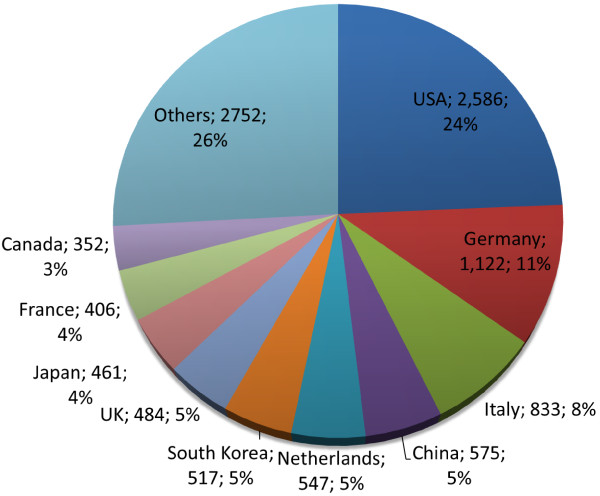
**The share of publications on stent-related research.**

**Figure 3:**
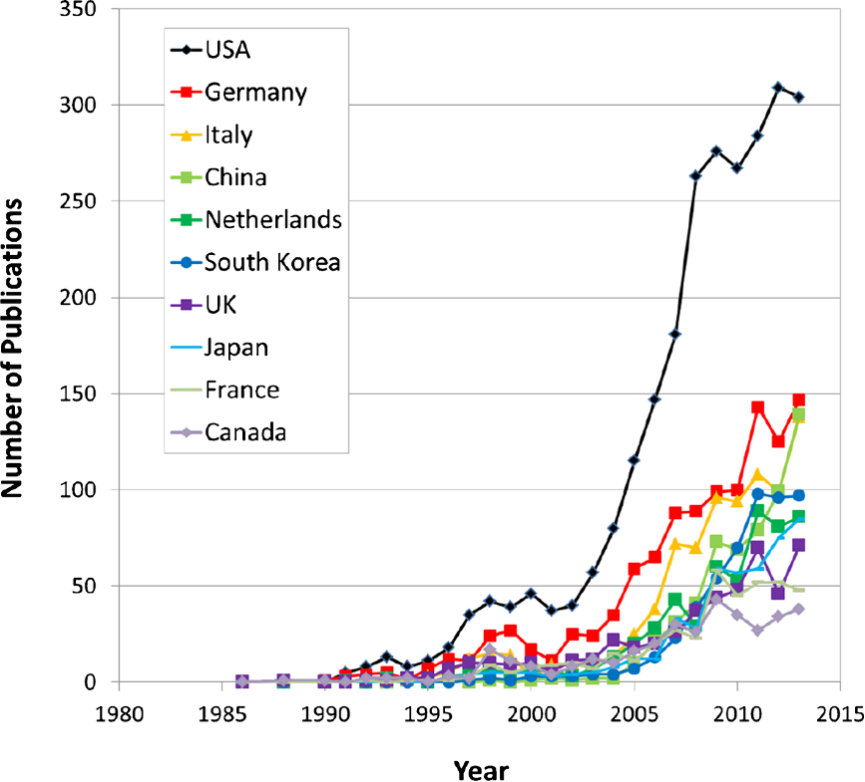
**Annual number of publications on stent by year of top-10 countries.**

1

where *L* is an upper limit of technology growth, *t* means time and the coefficients *α* and *β* are the parameters which determine the shape of the growth curves. We can estimate the year of inflection point to 2015.3 by the fitting curve as shown in the inset of Figure 1, which means the current research on stent being in the midst of active time.In order to investigate the stent-related research trends by the authors of countries, we have extracted county information from addresses of author-affiliation. The researches on stents have been performed by 71 countries from 1986 to 2013. Figure 2 shows a share of number of publications of top-10 countries which take a share about 74% of the total publications. USA ranks the first with 2,586 papers and Germany (1,122), Italy (833), China (575), Netherlands (547), South Korea (517), UK (484), Japan (461), France (406), and Canada (352) are come along after USA. The annual number of publications for the whole countries generally increases as shown in Figure 3.

The research performance should be considered quantitatively as well as qualitatively. If the simple number of publications is used as a quantitative indicator, the widely known indicator for research quality measurement is based on citation. Here, we have used the quality-factor(*Q-factor*), which is defined as the average number of citations per publication(*CPP*) of a country compared to the world-wide average *CPP* in a given research field [5]. It could be regarded that the research level is over world average level when the *Q-factor* is more than 1. The Netherlands shows 1.64 as the highest *Q-factor* and Canada (1.45), UK (1.39), France (1.31), USA (1.23), Italy (1.15) and Germany (1.14) show the values over 1.

**Figure 4:**
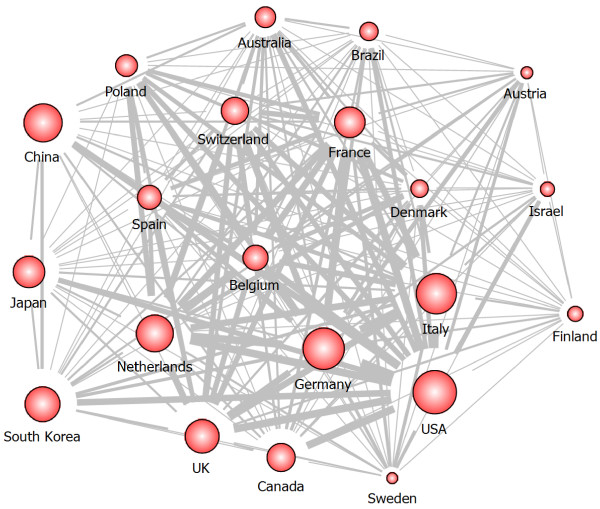
**Collaboration network map among top-21 countries.**

**Figure 5:**
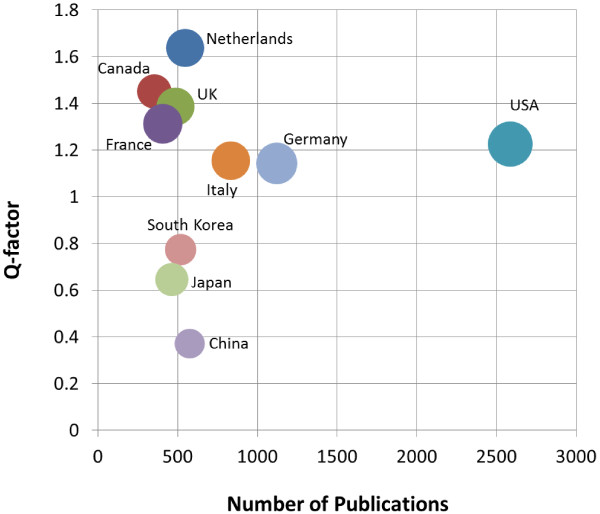
***Q-factor*** **vs. number of publications; the size of circles refers the number of collaborations.**

The collaboration network analysis between countries has been investigated. USA collaborates with 57 countries including Germany, Italy, the Netherlands, Canada, France, UK, and South Korea on stent-related research. Figure 4 shows the international collaboration network of top-21 countries publishing more than 100 papers. The width of lines means the strength of collaboration and the size of nodes represents the number of publications of the country. We can find that research on stent has been performed and developed by building strong collaboration between USA and other countries in Europe. Table 2 shows the summary of quantitative and qualitative indicator-values for top-10 countries of the stent-related research. Figure 5 shows the scatter chart to see the relation between Q-factor and number of publications and the size of circles refers the number of collaborations.

**Table 2:** **Quantitative and qualitative indicators for top-10 countries of the stent-related research**

Countries	No. of pub.	CPP	Q-factor	No. of collab.
**USA**	2586	23.24	1.23	57
**Germany**	1122	21.67	1.14	48
**Italy**	833	21.88	1.15	43
**China**	575	7.03	0.37	25
**Netherlands**	547	31.02	1.64	41
**South Korea**	518	14.69	0.77	28
**UK**	484	26.26	1.39	41
**Japan**	461	12.22	0.64	31
**France**	406	24.88	1.31	44
**Canada**	352	27.50	1.45	33

**Figure 6:**
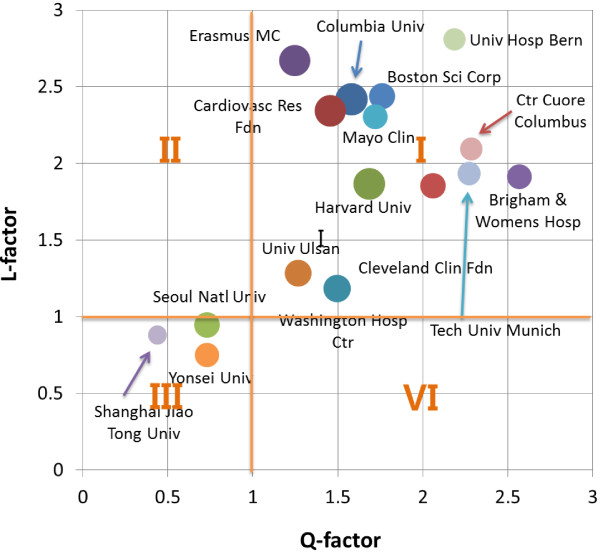
***L-factor*** **vs.** ***Q-factor*** **of top-20 institutions; the size of circles refers the number of publications.**

In a view of institution, about 5,000 institutions published the stent-related journal articles. Table 3 shows the status of top-20 institutions. Columbia University in USA published 214 papers and Cardiovascular Research Foundation (202), Harvard University (202), Erasmus University Medical Center (199) and Washington Hospital Center (153) published over 150 papers. Many of institutes are collaborating with each other for a research on stent. Thus, we have used the international collaboration strength (*ICS*) indicator which is obtained by ‘the share of foreign institutions collaborating with a certain institution in its total collaborating’. Similar to the *Q-factor*, the field-averaged *ICS* is defined as the L-factor. As a same rule for the *Q-factor*, an institution is said to be at the field average when its *L-factor* is equal to unit [5]. Figure 6 shows the relationship of *Q-L* values of the top-20 institutions. As shown in the Figure 6, the plot is divided into four sections by the guide-line (*Q* = 1 and *L* = 1). The institutions in the Section I have high quality and high international network where almost the whole top-20 institutions are belonging. The institutions in Section III (Seoul National University, Yonsei University and Shanghai Jiao Tong University) show low performance and relatively domestic network though large publication outputs.

**Table 3:** **The top-20 institutions in the field of stent research**

Institution	Country	No. of pub.	CPP	Q	L	Section
**Columbia Univ.**	USA	214	29.94	1.58	2.42	I
**Cardiovasc Res Fdn.**	USA	202	27.58	1.45	2.34	I
**Harvard Univ.**	USA	202	31.95	1.69	1.87	I
**Erasmus MC**	Netherlands	199	23.62	1.25	2.67	I
**Washington Hosp Ctr.**	USA	153	28.33	1.49	1.18	I
**Univ. Ulsan**	South Korea	146	23.99	1.27	1.28	I
**Boston Sci Corp.**	USA	136	33.36	1.76	2.44	I
**Cleveland Clin Fdn.**	USA	133	39.02	2.06	1.85	I
**Seoul Natl Univ.**	South Korea	132	13.87	0.73	0.95	III
**Brigham & Womens Hosp.**	USA	129	48.69	2.57	1.91	I
**Mayo Clin.**	USA	124	32.58	1.72	2.30	I
**Yonsei Univ.**	South Korea	119	13.82	0.73	0.75	III
**Tech Univ. Munich**	Germany	112	43.08	2.27	1.93	I
**Ctr.Cuore Columbus**	Italy	104	43.34	2.29	2.09	I
**Univ. Hosp Bern**	Switzerland	104	41.46	2.19	2.81	I
**Duke Univ.**	USA	102	37.61	1.98	1.65	I
**Stanford Univ.**	USA	101	24.95	1.32	2.20	I
**Mt Sinai Med Ctr.**	USA	98	24.31	1.28	2.24	I
**Univ. Toronto**	Canada	79	33.70	1.78	1.76	I
**Shanghai Jiao Tong Univ.**	China	75	8.31	0.44	0.88	III

**Figure 7:**
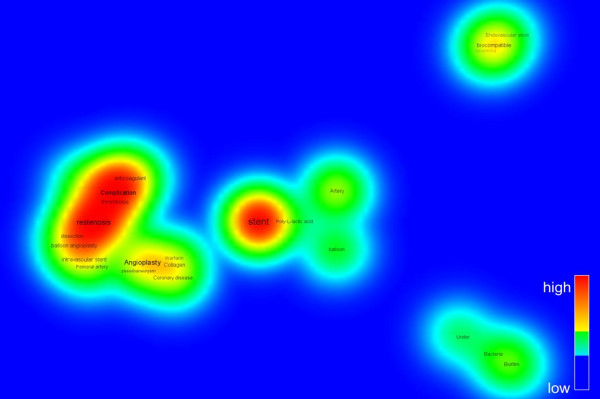
**Density view of keyword map in Period I (1986 ~ 1995); the red color corresponds the highest density and the blue color corresponds the lowest density.**

**Figure 8:**
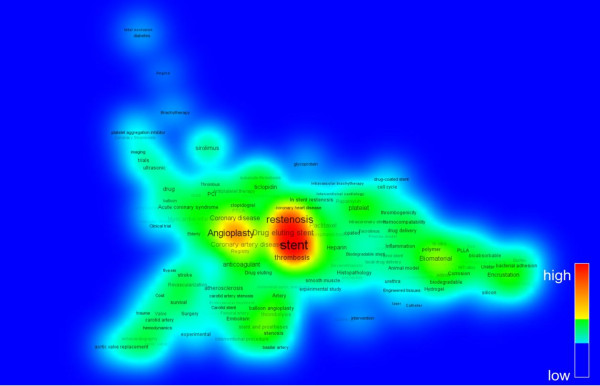
**Density view of keyword map in Period II (1996 ~ 2005).**

**Figure 9:**
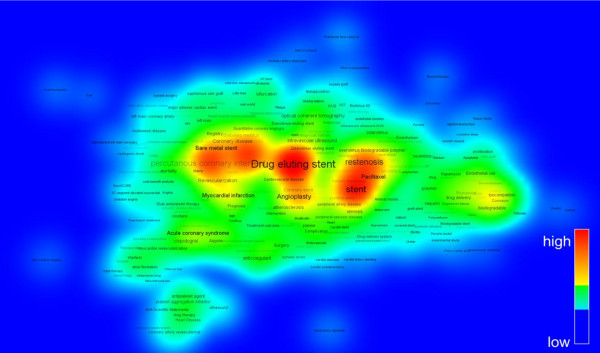
**Density view of keyword map in Period III (2006 ~ 2013).**

Meanwhile, the research trends can be understood in a view of topic changing. The visualization technique by the co-word mapping is widely used to identify knowledge structure in a certain research field. In the present study, keywords given by authors are used with the VOSviewer software, which suggests the combination algorithm of mapping and clustering by intuition. Here, the period is divided into 3 parts. Figures 7, 8 and 9 shows the density view of keyword map based on the co-occurrence matrix by periods. The color of the point in a map represents the density of the point depending on the number of neighboring items and on the weights of these items. The color of point with the larger number of neighboring items of a point and the higher weights of neighboring items is to be red. Consequently, the opposite case is to be blue. Also, the font size of the item’s label depends on the weight of the item which is determined by the appearance frequency. And the location of the words reflects the Euclidean distances between all pairs of items [6].

The Period I during first 10 years from 1986 to 1995 is the beginning stage. As shown in Figure 7, the words such as ‘stent’, ‘restenosis’, ‘biocompatible’ are appeared with a few frequencies. The Period II (1996 ~ 2005) could be regarded as a time of differentiation. Figure 8 shows that the number of keywords are rapidly increased and diversified as the advances of research on stent. The remarkable keywords are ‘coronary artery disease’, ‘drug eluting stent’, ‘thrombosis’, ‘inflammation’, ‘drug-coated stent’, and so on. Period III (2006 ~ 2013) is called as the time of expansion and stabilization. As seen in Figure 9, ‘drug eluting stent’ is emerging as a main topic of stent-related research and keywords such as ‘paclitaxel’, ‘sirolimus’, ‘drug delivery’, ‘zotarolimus’, ‘everolimus biodegradable polymer’, ‘endothelial function’ stand out as being representative words. We are able to know that many efforts are under way to overcome the present limitations of stent such as thrombosis. These keywords network analysis results correspond to the previous reports on development of stent-related research [7].

## Conclusions

Since the first stent was implanted in human coronary arteries by Puel and Sigwart in 1986, the advances of interventional treatment of coronary artery disease was remarkably progressed. It was results from the continuous development of the medical kits including stents, the accumulative experiences of operators and use of new antiplatelets [8]. Nowadays, stent coated with new polymeric drugs may have different activities in terms of affecting endothelium and vascular inflammation. Polymeric drug coating with NO-donors may decrease platelet adhesion and coagulation. Stent coated with polymeric CD34-antibodies may be able to prevent thrombosis by accelerating endothelial coverage and may capture circulating endothelial progenitor cells. Furthermore, development of biodegradable stents might also be useful way to decrease the incidence of late thrombosis on the stent. Anti-thrombotic therapy may be likely to be optimized with the development of more newly efficient anti-coagulant and anti-platelet drugs with a lower risk of bleeding complications [9, 10].

The research trends on stent for blood arteries are reviewed by bibliometric analysis using 7,790 journal articles of Web of Science database published from 1986 to 2013. We have applied the bibliometric indicators to analyze the journal article data, which contain simple number of publications for selecting key players, citation indicators for measuring qualitative research performance, collaboration indicators for figuring out the degree of international collaboration and keyword mapping for identifying the research trends. The stent-related research has been performed in 71 countries including USA, Germany, Italy, etc. The countries at the forefront published many high-quality journal articles with strong international collaboration. The top-20 institutions such as Columbia University, Cardiovascular Research Foundation, Harvard University, etc. are also investigated as the core institutions on stent-related research. On the basis of the keyword network maps as time period, the research trends in a view of topic changing are finally explored. The topics such as ‘drug eluting stent’, ‘stent coated with new polymers’ and ‘drug delivery systems’ are prominent lately, which means lots of efforts are under way to overcome the present limitations of stent-related research such as thrombosis and polymeric drug coatings. The future of stent is expected to be more perspective on developing of innovative ‘biodegradable/bioabsorbable’ polymer and stents.
